# Supplementary material for: Diet, exercise and mental-wellbeing of healthcare professionals (doctors, dentists and nurses) in Pakistan
Source: PeerJ. 2015 Sep 17;3:e1250. doi: 10.7717/peerj.1250 (PMC4579013; doi:10.7717/peerj.1250)
Supplement: Table S1 [file peerj-03-1250-s002.doc]

| **Appendix-1: Food Exchange List** | | |
| --- | --- | --- |
| **FOOD GROUP** | **COMPONENT** | **SERVING SIZE or EXCHANGE (E)** |
| **Starchy**  **Food** | Roti**^2^** | 1 Roti (8 inches)=2E |
|  | Paratha***^,2^** | 1 Paratha (8 inches)=2E |
|  | Rice**^1^** | 1 medium plate containing 1 cup cooked rice=3E |
|  | Breakfast Cereals**^1^** | 1 medium bowl=1.33E |
|  | Bread**^1^** | 1 Slice=1E |
| **Meat, Fish & other Protein Source** | Red Meat**^1^** | 1 medium sized pieces(Boti)=3E |
|  | Chicken**^1^** | 1 medium sized pieces(Boti)=3E |
|  | Fish**^1^** | 1 med size fish=3E |
|  | Eggs**^1^** | 1 medium sized=1E |
|  | Pulses(Dal, Pea)**^1^** | 1 medium plate containing 1 cup of pulses= 2E |
| **Dairy** | Milk**^1^** | 236ml=1E |
|  | Yogurt**^1^** | 177.4g=1E |
| **Fruits & Vegetables** | Large sized fruits (e.g. mango or pineapple)**^1^** | 2 slices=1E |
|  | Medium sized fruits (e.g. apple or banana)**^1^** | 1 medium sized fruit=1E |
|  | Small fruits (e.g. plums)**^1^** | 2 small fruits=1E |
|  | Grapes & Berries**^1^** | 2 handfuls=1E |
|  | Fruit juices**^1^** | 118 ml=1E |
|  | Vegetables**^1^** | 1 medium plate containing 1 cup cooked vegetables=3E |
|  | One medium=2E | Potato (Baked)**^1^** |
|  | Salad leaves**^1^** | 1 dessert bowl= 1E |

***= Contains fat**

**Sources:**

# Krause's Food & Nutrition Therapy (12^th^ Edition)

1. FSANZ Online database: NUTTAB as well as “The New Traffic Light Guide” by the Diabetes Education and Assessment Program, Royal North Shore Hospital, Australia.
